# Analysis of α- and β-amanitin in Human Plasma at Subnanogram per Milliliter Levels by Reversed Phase Ultra-High Performance Liquid Chromatography Coupled to Orbitrap Mass Spectrometry

**DOI:** 10.3390/toxins12110671

**Published:** 2020-10-23

**Authors:** Thomas P. Bambauer, Lea Wagmann, Armin A. Weber, Markus R. Meyer

**Affiliations:** Department of Experimental and Clinical Toxicology, Institute of Experimental and Clinical Pharmacology and Toxicology, Center for Molecular Signaling (PZMS), Saarland University, 66421 Homburg, Germany; thomas.bambauer@uks.eu (T.P.B.); Lea.wagmann@uks.eu (L.W.); armin.weber@uks.eu (A.A.W.)

**Keywords:** amatoxins, amanitin, mushroom poisoning, LC-HRMS/MS, bioanalysis

## Abstract

Amatoxins are known to be one of the main causes of serious to fatal mushroom intoxication. Thorough treatment, analytical confirmation, or exclusion of amatoxin intake is crucial in the case of any suspected mushroom poisoning. Urine is often the preferred matrix due to its higher concentrations compared to other body fluids. If urine is not available, analysis of human blood plasma is a valuable alternative for assessing the severity of intoxications. The aim of this study was to develop and validate a liquid chromatography (LC)-high resolution tandem mass spectrometry (HRMS/MS) method for confirmation and quantitation of α- and β-amanitin in human plasma at subnanogram per milliliter levels. Plasma samples of humans after suspected intake of amatoxin-containing mushrooms should be analyzed and amounts of toxins compared with already published data as well as with matched urine samples. Sample preparation consisted of protein precipitation, aqueous liquid-liquid extraction, and solid-phase extraction. Full chromatographical separation of analytes was achieved using reversed-phase chromatography. Orbitrap-based MS allowed for sufficiently sensitive identification and quantification. Validation was successfully carried out, including analytical selectivity, carry-over, matrix effects, accuracy, precision, and dilution integrity. Limits of identification were 20 pg/mL and calibration ranged from 20 pg/mL to 2000 pg/mL. The method was applied to analyze nine human plasma samples that were submitted along with urine samples tested positive for amatoxins. α-Amanitin could be identified in each plasma sample at a range from 37–2890 pg/mL, and β-amanitin was found in seven plasma samples ranging from <20–7520 pg/mL. A LC-HRMS/MS method for the quantitation of amatoxins in human blood plasma at subnanogram per milliliter levels was developed, validated, and used for the analysis of plasma samples. The method provides a valuable alternative to urine analysis, allowing thorough patient treatment but also further study the toxicokinetics of amatoxins.

## 1. Introduction

Intoxication by wild mushrooms can occur when edible species are misidentified or toxic species are ingested accidentally by children or used intentionally in the case of suicide attempts or homicides [1,2,3]. Some mushroom poisoning syndromes are potentially life-threatening [3,4,5]. Mushrooms that contain amatoxins especially have been known to be the main cause of severe to fatal mushroom poisoning for decades [6,7]. Recently, De Olano et al. reported a mortality rate of 8.8% of suspected amatoxin poisoning cases (*n* = 148) from 2008 to 2018 in the US, while studies from Portugal reported 11.8% (1990–2008; *n* = 93) and South China just under 20% (1994–2012; *n* = 624) [8,9,10]. This diversity could be explained by different strategies in intensive care and by endemic species in Asia containing higher amounts of amatoxins [10,11,12,13]. These thermally stable cyclic octapeptides can be found in *Amanita phalloides* and in many other *Amanita* species, as well as in certain species of the genera *Lepiota*, *Galerina*, and *Pholiotina* [11,13,14,15,16]. Once absorbed, amatoxins are rapidly excreted into bile and urine, and there is no evidence of metabolism, such as cleavage by proteases [13]. Thus, enterohepatic circulation, according to a study with perfused rat livers, is assumed to have a big impact on the prognosis [13,17]. After importing into hepatocytes via the organic anion transporting protein 1B3 (OATP1B3), amatoxins bind to RNA polymerase II in the cell nuclei with high affinity (nM range), leading to apoptosis [18,19]. The first signs of intoxication appear as gastro-intestinal symptoms 6–24 h (mostly 10–12 h) after the mushroom meal, whose delay is characteristic but not specific for amatoxin poisoning [13]. Nevertheless, slightly toxic or edible but rotten mushrooms cause most mushroom poisonings, and they can manifest in very similar moderate gastro-intestinal symptoms [1,20,21]. The first symptoms of acute hepatic injury are typically seen on the third day after ingestion. Over the following days, the poisoning can evolve into a serious hepatorenal syndrome with the potential failure of the liver and kidney, requiring a liver transplant or human albumin dialysis [13]. The most frequently used antidotal therapy options consist of penicillin G, *N*-acetylcysteine (NAC), and silibinin/silymarin. It is controversially debated, but a slight benefit for the latter in monotherapy may exist (Grade II-2 evidence) [6,13,22,23]. An early start of therapeutic and supportive treatment as well as the application of activated charcoal is essential to prevent the progression of the syndrome [13,22,24,25]. To allow thorough treatment, a fast exclusion or confirmation of amatoxin presence in human biosamples at an early stage of any suspected mushroom poisoning should be possible. Urine is usually the matrix of choice for such analysis due to higher concentrations compared to other body fluids [26]. Several methods were published for their analysis in urine by using LC-MS, LC-(HR)MS/MS, capillary zone electrophoresis, HPLC, radioimmunoassay, ELISA, and recently a lateral flow immunoassay [27,28,29,30,31,32,33,34,35,36,37]. Also, blood plasma or serum can be used as demonstrated earlier [33,34,38,39,40,41]. To our knowledge, only one study (in English) was published where the toxins were also found in human plasma or serum samples by using mass spectrometry [41]. Providing a limit of detection (LOD) of 200 pg/mL, Filigenzi et al. were able to identify α-amanitin by using mass spectrometry in two serum samples that had been taken within the first 24 h after ingestion, while three others, sampled after 24–48 h, were negative [41]. Usually, only trace amounts of amatoxins are circulating in blood after 24 h of ingestion. Thus, LODs of <200 pg/mL are needed to analyze for amatoxins in human plasma or serum, particularly when samples were taken >24h after ingestion. Although urine is the matrix of choice, human plasma/serum is a valuable additional tool, e.g., in cases when the sampling of urine is not possible. Moreover, analyzing for low amounts of amatoxins in the plasma or serum would allow us to monitor the effectiveness of secondary detoxification measures and to elucidate still unknown details of amatoxin toxicokinetics. 

Therefore, the aim of this study was to develop and validate a fast and sensitive liquid chromatography (LC)-high resolution mass spectrometry (HRMS/MS) method for confirmation and quantitation of α- and β-amanitin in human plasma. Plasma samples of humans after a suspected intake of amatoxin containing mushrooms are analyzed. The amounts of toxins are then compared with published data. Furthermore, results are compared with amounts of α-, and β-amanitin in matched urine samples, analyzed using a published method [42]. 

## 2. Results 

### 2.1. Method Development 

**Figure 1:**
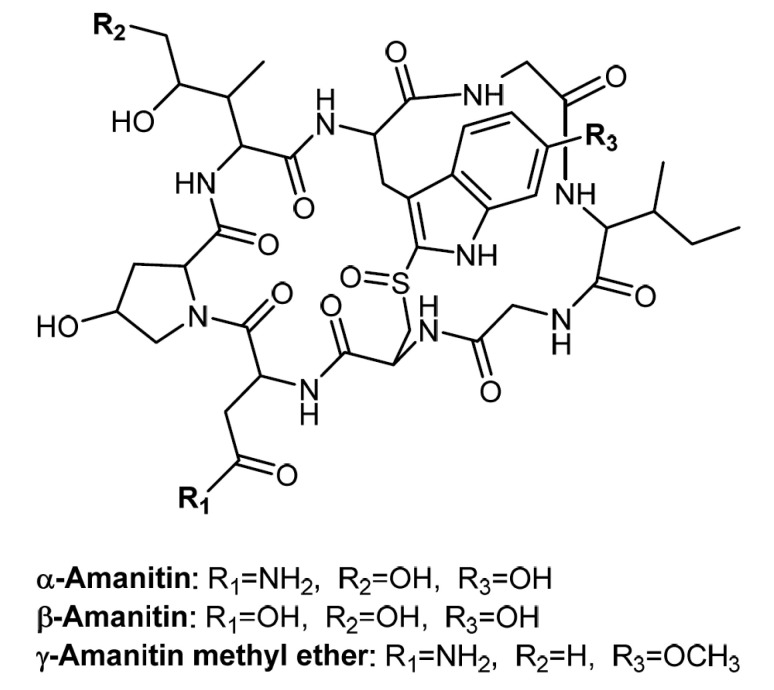
Structures of α- and β-amanitin and the internal standard of γ-amanitin methyl ether.

Sample preparation consisting of protein precipitation by acetonitrile, aqueous liquid-liquid extraction (A-LLE) by dichloromethane, and solid-phase extraction (SPE) was necessary to allow sufficient enrichment of analytes α-amanitin, β-amanitin, and the internal standard (IS) γ-amanitin methyl ether (Figure 1) as well as removal of the biomatrix. A similar strategy was published by Filigenzi et al. [41]. Before SPE was performed, the A-LLE extract had to be acidified because β-amanitin was only retained in the SPE column when in the protonated (uncharged) form. Strata X-Drug B cartridges (mixed-mode strong cation-exchange resin) and a similar SPE protocol had been used successfully in a previous study [30]. In the current study, we determined an extraction recovery of 50.2% (coefficient of variation (CV) = 11.4%), 55.7% (CV = 8.4%), and 87.6% (CV = 19.8%) for α-amanitin, β-amanitin, and γ-amanitin methyl ether (internal standard), respectively. Filigenzi et al. reported a significantly higher recovery of 95% (CV = 8.8%) for α-amanitin (recovery for β-amanitin not reported) in serum samples [41].

**Figure 2:**
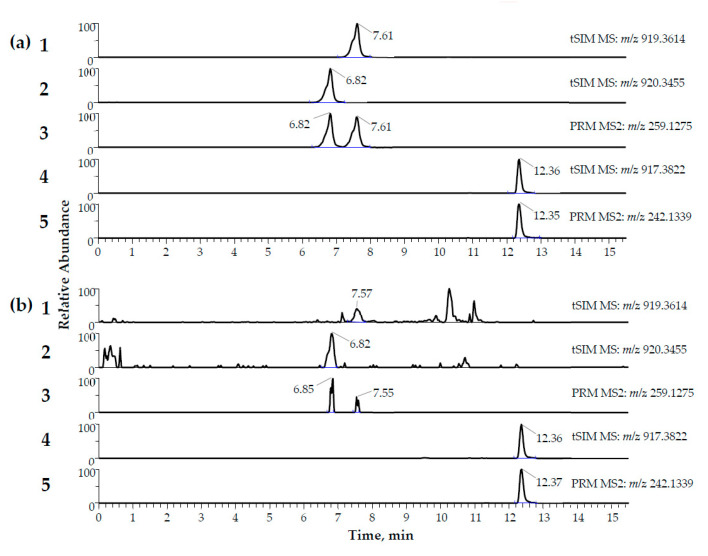
Extracted ion chromatograms (MH^+^) of matrix-matched quality control samples ((**a**) = 2000 pg/mL, (**b**) = 20 pg/mL) of α-amanitin (m/z = 919.3614 at 7.61 min in (**a**)**1** and 7.57 min in (**b**)**1**), β-amanitin (m/z = 920.3614 at 6.82 min in (**a**)**2** and (**b**)**2**), and the internal standard (IS) γ-amanitin methyl ether (m/z = 917.3822 at 12.36 min in (**a**)**4** and (**b**)**4**). The ion chromatograms of the characteristic fragment ions for the identification of α- and β-amanitin in MS^2^ are shown in (**a**)**3** and (**b**)**3**. The ion chromatograms of the characteristic fragment ions for the identification of the IS are shown in (**a**)**5** and (**b**)**5**. The mass deviation was set to 5 ppm.

High-resolution signals of protonated molecules (MH^+^) of α- and β-amanitin were used for quantification, and their specific parallel reaction monitoring (PRM) MS^2^ fragment ion at m/z 259.1275 allowed identification. Normalized collision energy (NCE) of 28% provided the highest abundance of this fragment ion. The best sensitivity in terms of LOIs was achieved when the isolation window was widened to m/z 4.5 for inclusion of α- and β-amanitin and their most common ^13^C-isotopes [43]. In order to discriminate α- from β-amanitin, baseline separation in LC was mandatory, which was achieved (Figure 2) by using an Accucore PhenylHexyl column and a gradient elution (Table 1). 

### 2.2. Method Validation 

No interfering signals were observed in the selectivity and analyte carry-over experiments. Matrix effects (ME) of the lower quality control (QC_Low_, 60 pg/mL) were 106% (CV = 12.0%), 109% (CV = 16.6%), and 102% (CV = 9.0%) for α-amanitin, β-amanitin, and the IS, respectively. MEs at QC_High_ samples (1600 pg/mL) were 102% (CV = 3.4%), 104% (CV = 3.7%), and 91.0% (CV = 13.7%) for α-amanitin, β-amanitin, and the IS, respectively. 

Amatoxins at concentrations of 20 pg/mL could be reliably identified across all runs and at several days (Figure 2). A linear regression, weighted by 1/x^2^, was used for quantification of α- and β-amanitin. In 6 calibrations on different days, mean R^2^ was 0.9973 (SD = 0.0013). Every single R^2^ for each calibration was >0.9950. The results of all accuracy, precision, and dilution integrity experiments are summarized in Table 2 and Table 3. In brief, all results matched the acceptance criteria. The dilution integrity allowed us to determine concentrations above the calibration range up to 100,000 pg/mL. The accuracy and precision of a 1:2 dilution were tested for cases when less sample material might be available for analysis. It should be mentioned that limits of identification (LOI) and lower limits of quantification (LLOQ) are increased by factor 2 in these cases.

### 2.3. Applicability

The results of the analyzed plasma samples and matched urine samples are listed in Table 4. In summary, α-amanitin could be identified in all nine plasma samples, β-amanitin only in seven. According to the reports by Tang et al. and Sgambelluri et al., in a few species of the genera *Amanita* and *Lepiota*, α-amanitin but no β-amanitin might be found [11,44]. Regarding both negative results in plasma, it is unlikely that β-amanitin was completely absent, as it may be present in concentrations below the LOI, since it was identified in the corresponding urine samples. Concentration of α-amanitin in plasma varied from 37 to 2890 pg/mL and β-amanitin from <20 to 7520 pg/mL. 

## 3. Discussion

Exclusion or confirmation of an intake of amanitin containing mushrooms using α- and β-amanitin as biomarkers is often requested to avoid expensive and time-consuming treatment of every suspected case. The sufficient sensitivity of such an analysis is crucial as hospitalization is often late after intake and only trace amounts of toxins can be found [26,27]. Therefore, urine is considered as the biomatrix of choice, as concentrations of amatoxins are usually higher here than in serum or plasma [26,45]. The major drawbacks of urine are a reduced output in the case of decreased renal function and acute renal failure, occurring in some amatoxin but also other mushroom poisoning cases, and a higher intra- and interindividual variability in the complexity of urine as biomatrix. If therapy measures like fluid replacement or forced diuresis have been conducted, low amounts of amatoxins in urine could have been further diluted. Some authors also reported high matrix effects, particularly when using positive electrospray ionization [35,42]. Therefore, a method for the determination of amatoxins in blood plasma may overcome some of these limitations. However, such a method requires sufficient sensitivity. According to Busi et al., concentrations in plasma should be about 10 times lower than in urine (without considering urinary creatinine for normalization) [34,46]. A reliable and sufficiently powerful method for amatoxin analysis in plasma would therefore be an important tool not only for acute clinical purposes but also in forensic toxicology and further research on toxicokinetics of amatoxins. To the best of our knowledge, there is currently no published method, which might support existing methods for urine analysis in terms of relative sensitivity and, thus, usefulness in an analytical setting.

### 3.1. Method Development 

γ-Amanitin methyl ether was used as an internal standard (IS) because of the similar structure (Figure 1), extraction, and elution behavior (Figure 2) [35,42]. Luo et al. published the production of ^15^N_10_-α-amanitin that already has been successfully used by Abbott et al. as an IS for their LC-MS/MS method [29,47]. Unfortunately, this or other structure-analog compounds of α- or β-amanitin that can be used as internal standards are currently not commercially available. 

### 3.2. Method Validation 

In summary, the matrix effects were very low compared to reported MEs in urine samples [29,42,48]. The method by Abbott et al. especially suffered from high matrix effects for α- (25.5% ± 2.50%) and β-amanitin (38.1% ± 1.29%), determined in pooled urine samples [29].

According to the ICH M10 guideline draft, several stability studies should be examined [49]. Li et al. provided the following stability data of α-amanitin in plasma: short-term, long-term, freeze-thaw (3 cycles), and postpreparative storage stability (*n* = 6; 3 concentration levels evaluated for each experiment) [50]. In addition, Zhang et al. also included β-amanitin in their stability studies in plasma, serum, and urine (*n* = 3; after 1, 2, 4, and 8 weeks) [33]. Maurer et al. provided most of the required stability data for the toxins and the IS γ-amanitin methyl ether as well as long-term stability in stock solutions for 6 months [35]. In none of these studies could any instability or decomposition exceeding 10% loss of recovery of α-, β-amanitin, or the IS be detected.

### 3.3. Applicability

Plasma concentrations shown in Table 4 were in line with the findings of Filigenzi et al. that were already mentioned, but differed from the results of the most comprehensive study of amatoxins in humans by Jaeger et al., who reported values in plasma ranging from 8000 to 190,000 pg/mL and from 23,500 to 162,000 pg/mL for α-and β-amanitin, respectively [26,41]. However, Jaeger et al. could detect amatoxins only in 11 of a total of 43 human plasma samples, of which 40 were taken between 18 and 96 h after the ingestion of mushrooms. They used a not validated (e.g., no data on selectivity) HPLC-UV method, based on a publication by Jehl et al. in 1985 with LODs for each toxin of 5000 pg/mL [26,51].

**Figure 3:**
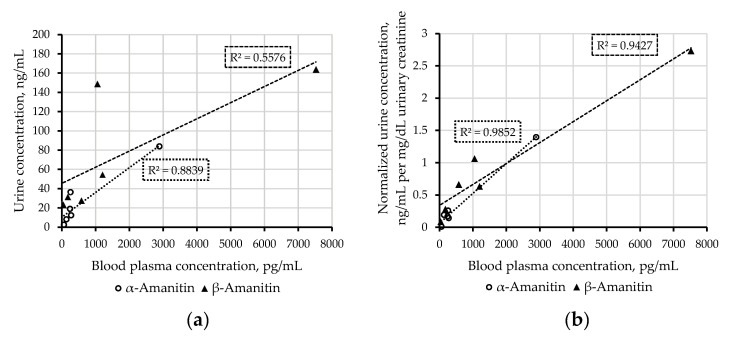
(**a**) Urinary concentrations (ng/mL) of α-and β-amanitin in patient case samples plotted against concentrations (pg/mL) in matched blood plasma samples; (**b**) creatinine-normalized urinary concentrations ((ng/mL)/(mg/dL)) of α-and β-amanitin in patient case samples plotted against concentrations (pg/mL) in matched blood plasma.

**Figure 4:**
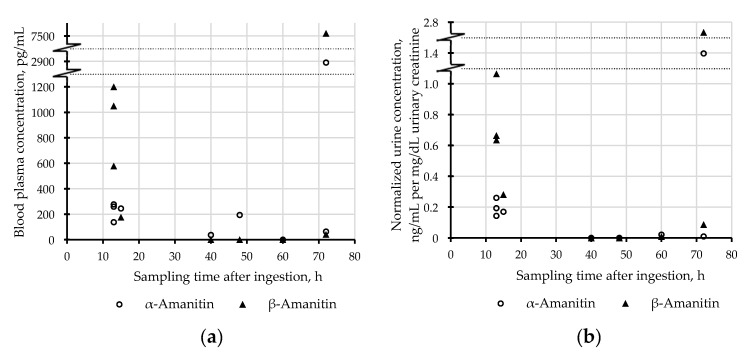
(**a**) Plasma concentrations (pg/mL) of α-and β-amanitin in patient case samples plotted against sampling time after the ingestion of toxic mushrooms; (**b**) creatinine-normalized urinary concentrations ((ng/mL)/(mg/dL)) of α-and β-amanitin in patient case samples plotted against sampling time after the ingestion of toxic mushrooms.

Figure 3 illustrates the correlation of plasma concentrations with uncorrected (Figure 3a) and creatinine-normalized urinary concentrations (Figure 3b). Creatinine-normalized urinary concentrations showed a better linear correlation and may thus be more comparable with plasma concentrations and therefore more suitable for interpretation than uncorrected ones. Furthermore, the effects of urine dilution, e.g., caused by therapy measures such as forced diuresis, can be normalized. This in turn allows us to compare results with creatinine normalized values of other studies. Thus, concentrations of amatoxins in urine should be published together with urinary creatinine. The time window of sampling after ingestion of the mushroom meal varied from 13 to 72 h. It must be kept in mind that this information, as well as the suspected amounts of ingested mushrooms, is based on patient information, and should be taken with care. A rapid decrease of toxin concentrations in both matrices can be observed when comparing values from samples collected after 13–15 h to samples collected after more than 40 h (Figure 4). It would be interesting to have data from more samples, particularly sampled before 13 h and between 15 and 40 h in order to make further conclusions. The determined concentrations of amatoxins in samples of case 9 seemed to be comparatively high, especially, when the late sampling time of approximately 72 h is considered. This patient suffered already from multi-organ failure when the samples were taken and shortly died afterward before liver transplantation could be realized. Such uncommon early deaths in the course of amatoxin poisoning were also described by Zilker and Faulstich [13]. For therapeutic reasons, it would be interesting to know, whether late-arising or persisting high concentrations of amatoxins in the blood may indicate a multi-organ failure.

## 4. Conclusions

A sufficiently sensitive and rather fast analytical method for the determination of α- and β-amanitin down to 20 pg/mL in blood plasma was developed, validated, and applied to analyze patient samples. Analysis showed that such sensitivity is needed for the reliable identification of these biomarkers. The presented method is an alternative in cases when urine as a biomatrix is not available. Furthermore, this method may allow us to answer still unsolved questions in toxicokinetics of amatoxins and could be applied to monitor plasma concentrations during therapy in order to evaluate the effectivity of secondary detoxification measures.

## 5. Materials and Methods 

### 5.1. Chemicals and Reagents

α- and β-amanitin with purity of ≥90% and ~90%, respectively, were purchased from Sigma-Aldrich (Taufkirchen, Germany) and γ-amanitin methyl ether was donated by Prof. Dr. Heinz Faulstich (Max-Planck-Institute for Cell Biology, Ladenburg, Germany). Methanol (MeOH), acetonitrile (ACN), dichloromethane (DCM), formic acid, and other chemicals were of analytical grade or better and were purchased from VWR (Darmstadt, Germany). All chemicals used for the preparation of eluents for LC-MS were of LC-MS grade except for acetic acid and ascorbic acid. Water was purified using a Milli-Q water purification system (Merck KGaA, Darmstadt, Germany) to reach a resistivity of 18.2 MΩ∙cm.

### 5.2. Stock Solutions for Calibration and Control Samples

Two lots of stock solutions were prepared by dissolving α- and β-amanitin at a concentration of 1000 µg/mL in MeOH for the preparation of stock solutions for calibration and control samples, respectively. Six levels of methanolic calibration stock solutions were prepared via dilution series with concentrations of α- and β-amanitin as follows: 50,000, 37,500, 25,000, 12,500, 2500, and 500 pg/mL. Five levels of methanolic stock solutions for QC samples were prepared: 50,000, 40,000, 10,000, 1500, and 500 pg/mL. A solution of γ-amanitin methyl ether at 2.5 µg/mL was prepared using a 1:1 mixture of purified water and MeOH. This solution was further diluted to 12,500 pg/mL in MeOH. In the following, 100 µL of this solution was added to each sample as an IS. All of the above-mentioned solutions were stored at +4 °C.

### 5.3. LC-HRMS/MS Apparatus

A Thermo Fisher Scientific Dionex UltiMate 3000 RS UHPLC system (Dreieich, Germany) was used with a quaternary UltiMate 3000 RS pump and an UltiMate WPS-3000 RS autosampler. The system and all its components were controlled by Thermo Fisher Chromeleon software version 6.80 SR11 (Thermo Fisher Scientific, San Jose, CA, USA). The LC system was coupled to a Thermo Fisher Q-Exactive Plus, equipped with a heated electrospray ionization II source (HESI-II). The conditions of HESI-II were adopted from Helfer et al. [52]: sheath gas, 60 arbitrary units (AU); auxiliary gas, 10 AU; spray voltage, 4.00 kV; heater temperature, 320 °C; ion transfer capillary temperature, 320 °C; and S-lens radio frequency (RF) level, 60.0%. External mass calibration was conducted as recommended by the manufacturer. Targeted selected ion monitoring (t-SIM) experiments were performed using the following scan parameters: General: runtime, 0 to 15.5 min, polarity, positive; in-source collision-induced dissociation (CID), 0.0 eV; inclusion, on; SIM: microscans, 1; resolution, 70,000; automatic gain control (AGC) target, 5e4; maximum injection time (IT), 200 ms; multiplex (MSX) count, 1; isolation window, 4.5 *m/z*; isolation offset, 0.0 *m/z*; spectrum data type, profile. Simultaneously, PRM experiments were performed for unambiguous identification of the analytes via specific mass fragments in MS^2^ scans. The PRM settings were as follows: General: runtime, 0 to 15.5 min, polarity, positive; in-source CID, 0.0 eV; dynamic retention time (RT), off; default charge state, 1; inclusion, on; MS^2^: microscans, 1; resolution, 70,000; AGC target, 2e5; maximum IT, 100 ms; loop count, 1; MSX count, 1; MSX isochroneous, on; isolation window, 4.5 *m/z*; isolation offset, 0.0 *m/z*; first fixed mass 80.0 *m/z*; NCE in high collision dissociation (HCD) cell, 28%; spectrum data type, profile. An inclusion list containing the exact masses of protonated, positively charged analytes (MH^+^) was used: α-amanitin (*m/z* 919.3614), β-amanitin (*m/z* 920.3455), γ-amanitin (*m/z* 903.3665), and γ-amanitin methyl ether (*m/z* 917.3822). The customized tolerance of mass deviations for this inclusion list was set to 10 ppm. 

### 5.4. Chromatography Setup

Gradient elution was performed on a Thermo Fisher Accucore PhenylHexyl column (150 mm × 2.1 mm I.D., 2.6 μm particle size) (Thermo Fisher Scientific, San Jose, CA, USA), a reversed-phase core-shell particle-based column, constantly heated at 40 °C. Eluent A was purified water, buffered with 8 mM ammonium acetate, acidified with 0.05% (*v/v*) acetic acid, and stabilized with 5 ppm ascorbic acid. Eluent B consisted of ACN and MeOH in equal shares, with 1% (*v/v*) purified water, 4 mM ammonium acetate, and 0.05% (*v/v*) acetic acid. The elution profile is shown in detail in Table 1. The total chromatography run time was 15.5 min. 

### 5.5. Analysis Raw Data Handling and Processing

Thermo Fisher Xcalibur software version 2.2 (Thermo Fisher Scientific, San Jose, CA, USA) was used for data handling and processing. The mass tolerance for peak integration was set to 5 ppm. ICIS peak integration settings were: smoothing points, 13; baseline window, 100; area noise factor, 2; and peak noise factor, 5.

### 5.6. Sample Preparation

The concept of sample preparation, consisting of protein precipitation, A-LLE, and SPE, was inspired by Filigenzi et al. [41]. Briefly, a volume of 2500 µL blood plasma was fortified with 100 µL of the IS solution. Defined levels of matrix-matched calibration and control samples were prepared by adding 100 µL of IS and stock solution to 2400 µL of human blank blood plasma.

#### 5.6.1. A-LLE

A volume of 6500 µL ACN was added for the precipitation of proteins. The mixture was then shaken for 2 min and centrifuged for 2 min using 3000 rotations per minute (1026× *g*), before the supernatant was decanted into another flask. After the addition of 11,000 µL DCM to this supernatant, shaking and centrifugation were repeated. A volume of 2500 µL of 0.1% formic acid was carefully added and the aqueous supernatant was transferred into a glass vial. 

#### 5.6.2. SPE

SPE was performed using Stata X-Drug B 33 µm Polymeric Strong Cation cartridges (Phenomenex, Aschaffenburg, Germany), containing 60 mg of sorbent mass and 3 mL capacity. The SPE column was preconditioned and equilibrated with 2 × 1000 µL of MeOH and 1000 µL of 0.1% formic acid, respectively. The A-LLE extract was further diluted with 5 mL of 0.1% formic acid and 1000 µL of 1% formic acid before it was loaded onto the column. Then, the columns were washed in two steps, using 1000 µL of 1% formic acid and 2 × 1000 µL of 0.1% formic acid, respectively. The elution of analytes was induced by using 2 × 600 µL of MeOH. The obtained eluate was gently evaporated at 75 °C under a stream of nitrogen and subsequently reconstituted by only 20 µL of MeOH. Finally, a sample extract volume of 5 µL was injected into the LC system.

### 5.7. Method Validation

Validation was planned and conducted according to the draft version of ICH Guideline on bioanalytical method validation (EMA/CHMP/ICH/172948/2019) [49]. Validation experiments included selectivity, LOIs, analyte carry-over, MEs, calibration curve, and range, including lower and upper limits of quantification (LLOQ, ULOQ), accuracy and precision (inter- and intra-day), as well as dilution integrity. The same pool of blank matrix samples was used across all validation experiments except for selectivity and ME experiments. 

Selectivity was tested by analyzing a batch of 10 blood plasma samples from different donors in order to screen for potentially interfering signals from endogenous compounds. 

Carry-over was checked by running zero samples (matrix blank samples spiked with IS) after a spiked sample containing analyte concentrations at the ULOQ. The final concentrations of α- and β-amanitin in the injected extract were 250,000 pg/mL.

MEs were determined at low and high concentrated control samples (QC_low_ = 60 pg/mL, QC_high_ = 1600 pg/mL). Experiments were performed using six lots of pooled blank plasma samples (5 or 6 sources of plasma each lot), processed in triplicate. MEs were calculated for each analyte and the IS by dividing peak areas of the analytes spiked into plasma samples by those in neat standards. In addition, analyte and IS recovery at QC_high_-level (1600 pg/mL) was determined by dividing peak areas of the analytes spiked into plasma samples before extraction by those of the extracts of blank samples spiked afterward. Calculations were performed using Microsoft Excel 2016. MEs leading to deviations of <15% and CVs of <15% were regarded as acceptable [49].

LOIs were determined by analyzing spiked samples in triplicate. LOIs were set at the lowest concentration where the analytes could be identified in each of the samples and in a repeated analysis on another day. The definition of identification was based on Helfer et al. [52,53]. At the retention time of the analyte, the accurate mass of the precursor ion must be detected and a specific fragment of the corresponding high-resolution MS/MS spectrum must be present. The MS/MS fragment at *m/z* 259.1275 was considered as specific for the identification of α- or β-amanitin.

Final concentrations of calibrators were at 20, 100, 500, 1000, 1.500, and 2000 pg/mL and 6 full sets of calibration samples, prepared as described in Section 5.2 and Section 5.6, were analyzed on six different days. Calibration curves were calculated by using regression and weighting models, implemented in Xcalibur software version 2.2, on the ratios of full scan MS peak areas of the analytes and the IS. 

The accuracy and precision were evaluated by analyzing four levels of QC samples at 20, 60, 400, and 1600 pg/mL, all prepared as described in Section 5.2 and Section 5.6. For the calculation of concentrations and errors, each run of the QC samples contained a full calibration. For intra-day accuracy and precision, 5 repetitions of QC samples were analyzed that ran within a day. Inter-day accuracy and precision were evaluated by including repetitions of the mentioned intra-day experiments on two other days. Both errors of accuracy and precision should not exceed 15%, except for LLOQ samples (20%) [49].

Dilution integrity was tested for a 1:50 dilution of the highest calibrator (2000 pg/mL). Additionally, a 1:2 dilution was tested using the same calibrator. Intra-day accuracy and precision were evaluated for these diluted samples as described above. Errors of accuracy and precision should not exceed 15%, as for other QC samples [49].

### 5.8. Applicability

The applicability was tested by analyzing nine plasma samples after suspected intake of amanitin containing mushrooms. Amatoxin poisoning had already been confirmed by previous analyses of the corresponding urine samples [35,42]. Samples had been sent to the authors’ laboratory for toxicological analysis and were kept frozen at −20 °C. The urine samples were again analyzed [42]. Creatinine was determined using the P.I.A.^2^ device from Protzek (Lörrach, Germany).

## Figures and Tables

**Table 1:** The elution profile. The steps are ramping steps for the eluent ratio (%) and flow rate (mL/min).

Step	Time, min	Duration, sec	Flow Rate, mL/min	Eluent Ratio	
				A ^a^	B ^b^
**1**	0.00	60	0.30	99.0	1.0
**2**	1.00	120	0.30	99.0	1.0
**3**	3.00	420	0.20	85.0	15.0
**4**	10.00	150	0.20	70.0	30.0
**5**	12.50	90	0.20	1.0	99.0
**6**	14.00	0	0.30	1.0	99.0
**7**	14.00	89	0.30	99.0	1.0
**8**	15.48	1	0.30	99.0	1.0
**9**	15.50	0	0.05	99.0	1.0

^a^ eluent A: water, ammonium acetate 8 mM, acetic acid 0.05% (*v*/*v*), 5 ppm ascorbic acid, ^b^ eluent B: acetonitrile 49.5% (*v*/*v*), methanol 49.5% (*v*/*v*), water 1% (*v/v*), ammonium acetate 4 mM, acetic acid 0.05% (*v*/*v*).

**Table 2:** Intra-day accuracy, precision, and dilution integrity 1:2 and 1:50 of α- and β-amanitin (α, β). Calculated values are given with their standard deviations.

Intra-Day (*n* = 5)	Nominal Conc., pg/mL		Calculated Conc., pg/mL		Accuracy, %		Precision, %	
	α	β	α	β	α	β	α	β
**QC1 (LLOQ)**	20	20	19.5 ± 2.17	19.1 ± 2.22	−2.7	−4.5	11	12
**QC2 (low)**	60	60	62.0 ± 5,76	53.0 ± 3.89	3.4	−12	9.3	7.3
**QC3 (mid)**	400	400	421 ± 28.4	411 ± 27.4	5.3	2.8	6.7	6.7
**QC4 (high)**	1600	1600	1510 ± 23.4	1570 ± 15.5	−5.4	−1.6	1.6	1.0
**QC Dil F2 ^a^**	1000*2	1000*2	2140 ± 81.3	2065 ± 103	6.8	3.2	3.8	5.0
**QC Dil F50 ^b^**	40*50	40*50	1940 ± 130	1757 ± 157	−3.1	−12	6.7	8.9

^a^ diluted QC sample of 2000 pg/mL α- and β-amanitin, dilution factor: 2 ^b^ diluted QC sample of 2000 pg/mL α- and β-amanitin, dilution factor: 50.

**Table 3:** Inter-day accuracy and precision of α- and β-amanitin (α, β). The calculated values are given with their standard deviations.

Intra-Day (*n* = 5), 3 runs	Nominal conc., pg/mL		Calculated conc., pg/mL		Accuracy, %		Precision, %	
	α	β	α	β	α	β	α	β
**QC1 (LLOQ)**	20	20	21.0 ± 2.10	19.9 ± 2.72	4.9	−0.4	10	14
**QC2 (low)**	60	60	64.2 ± 4.92	55.8 ± 5.60	6.9	−7.0	7.7	10
**QC3 (mid)**	400	400	409 ± 24.2	401 ± 25.0	2.2	0.3	5.9	6.2
**QC4 (high)**	1600	1600	1514 ± 111	1535 ± 107	−5.4	−4.1	7.4	7.1

**Table 4:** Analysis results of blood plasma and urine samples of suspected mushroom poisoning cases.

Case		1	2	3	4	5	6	7	8	9
**Amounts of mushrooms ingested**		3–4	2–3	2–3	unk.	3	1 cap	unk.	unk.	unk.
**Sampling time after intake ^a^, h**		13	13	13	40	48	15	48-72	72	72
**Blood plasma concentration, pg/mL**	α-Amanitin	277	259	137	37	194 ^b^	245	<40 ^b^	63	2890
	β-Amanitin	1200	1050	578	<20	n.i. ^b^	176	n.i. ^b^	41	7520
**Urinary concentration, ng/mL**	α-Amanitin	12	36	8.1	n.i.	<1	19	4.6	2.5	24
	β-Amanitin	55	149	28	n.i.	<1	32	2.0	23	164
**Urinary creatinine, mg/dL**		86	140	42	169	232	112	220	268	60

^a^ time of ingestion is based on anamnestic data, ^b^ reduced amount of blood plasma (1.25 mL) utilized for quantification. Abbreviations: unk. = unknown; n.i. = not identified
